# A rare case of persistent lateral marginal vein of Servelle in Klippel Trenaunay Syndrome: A successful surgical management

**DOI:** 10.1016/j.ijscr.2022.107052

**Published:** 2022-04-06

**Authors:** S. Dahal, R.M. Karmacharya, S. Vaidya, K. Gautam, S. Bhatt, N. Bhandari

**Affiliations:** Department of Surgery (CTVS), Dhulikhel Hospital, Kathmandu University Hospital, Nepal

**Keywords:** Case report, Klippel-Trenaunay Syndrome, Port-wine stain, Radiofrequency ablation, Varicose veins

## Abstract

**Introduction and importance:**

Klippel-Trenaunay Syndrome (KTS) is a rare congenital vascular disorder characterized by capillary malformation, varicosities, and tissue overgrowth. It usually affects the unilateral lower extremities manifesting commonly as pain, localized rise of temperature, and venous tortuosity. However, in severe cases, ulceration, cellulitis, and chronic lymphatic malformation may be present. Management is mostly supportive and involves the use of compression stockings.

**Case presentation:**

Here, we report a case of KTS with a persistent lateral marginal vein of Servelle managed with radiofrequency ablation along with sclerotherapy of selected perforators. On a two-year follow-up, the symptoms had resolved and Doppler ultrasonography revealed resolution of the defective vein along with the absence of incompetent perforators.

**Clinical discussion:**

In cases with venous malformation with the persistence of embryonic avalvular venous structures, like the lateral marginal vein of Servelle, surgical intervention is warranted especially at a younger age to reduce the risk of future thromboembolic events and recurrence.

**Conclusion:**

Varicosities of the lateral marginal vein of Servelle can be managed successfully by radiofrequency ablation and adjunct sclerotherapy in selected cases.

## Highlights

• Klippel-Trenaunay Syndrome (KTS) is a congenital vascular disorder which usually affects the unilateral lower extremities.
• Endovenous treatment of the greater saphenous vein is gradually becoming popular in the treatment of KTS.
• This case is managed by radiofrequency ablation of lateral marginal vein of Servelle.

## 1 Introduction

Klippel-Trenaunay Syndrome (KTS) comprises a triad of capillary malformations, soft tissue and bone hypertrophy, and atypical varicose veins especially involving lateral marginal veins [1], [2]. This condition was described in 1900 by Maurice Klippel and Paul Trenaunay [1], [2]. Embryonic lateral marginal vein of Servelle is a common occurrence in this syndrome and is often associated with varicose veins which were first described in 1984 in a series of 768 cases [3].

Some of the indications of surgical treatment of venous malformation in KTS are significant cosmetic issues, pain, heaviness, and bleeding. If the disease primarily involves the superficial system or lateral marginal vein then venous stripping, ligation, excision, or sclerotherapy can be done [2]. Endovenous treatment options like laser ablation or radiofrequency ablation of the greater saphenous vein are gradually becoming popular in selected cases [2], [4]. Here we are reporting a case of KTS managed by radiofrequency ablation of lateral marginal vein of Servelle along with sclerotherapy of selected perforators. This case report is reported in accordance with SCARE 2020 Guidelines [5].

## 2 Case details

**Fig. 1:**
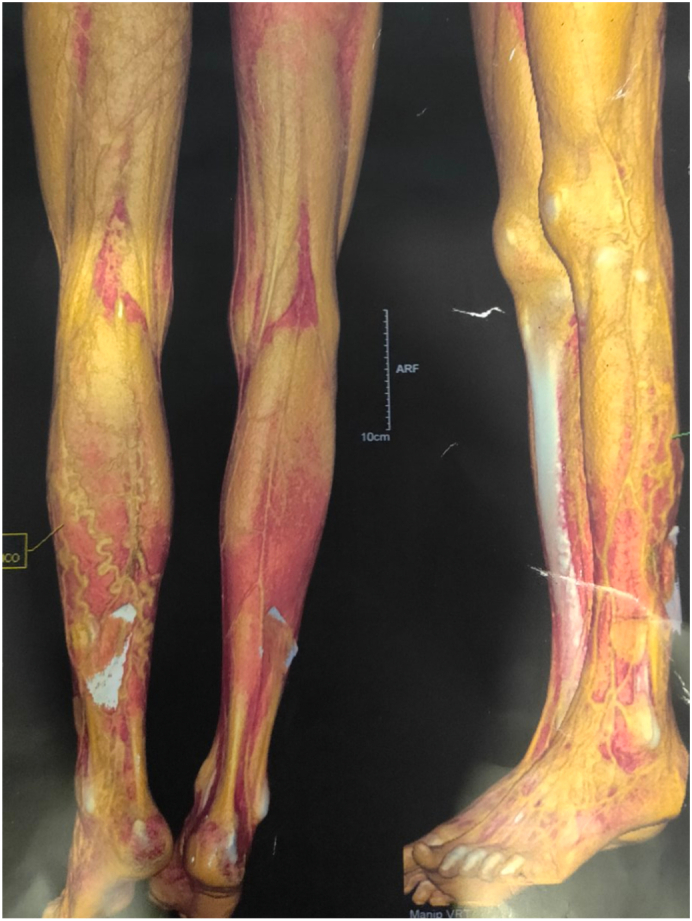
3D reconstruction of the abnormal veins in the lateral and posterior aspect of left thigh and calf.

A nineteen-year-old male presented with prominent dilated veins in his left leg since childhood which was initially not bothering him, but since one year he had been having swelling and pain in his left calf and leg. For six months, he had noticed some purplish pigmentation in the left calf and foot but without ulceration. There was however no history of trauma or surgical procedures done in the left lower limb. Doppler ultrasonography was done which showed dilated veins in the lateral aspect of the left lower limb starting from the ankle region and continuing up to the upper thigh. The maximum diameter of the vein was 7.3 mm. There was one long vein with multiple perforators between it and the deep veins and muscular veins. Deep veins were patent. These features are typical of the lateral marginal veins of Servelle. Computed Tomography Venogram reconfirmed the finding and 3D reconstruction showed the venous network and abnormal perforators in detail as shown in Fig. 1. The patient was advised for physiotherapy and compression stocking.

**Fig. 2:**
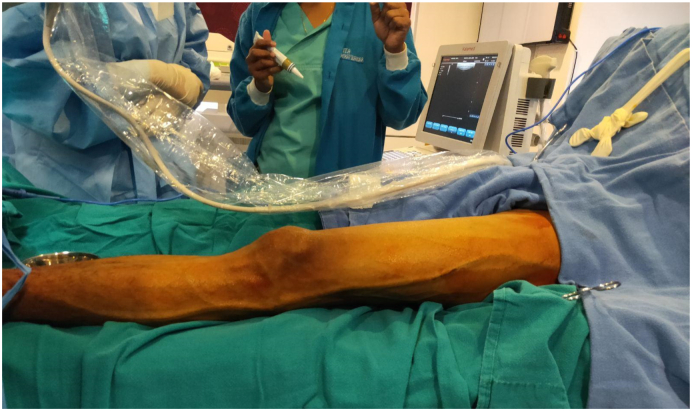
Pre radiofrequency ablation picture of lateral marginal vein of Servelle.

**Fig. 3:**
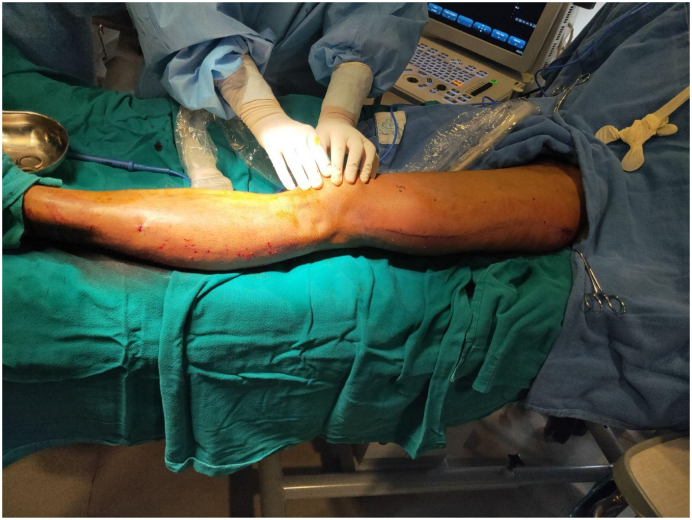
Post radiofrequency ablation picture of lateral marginal vein of Servelle.

**Fig. 4:**
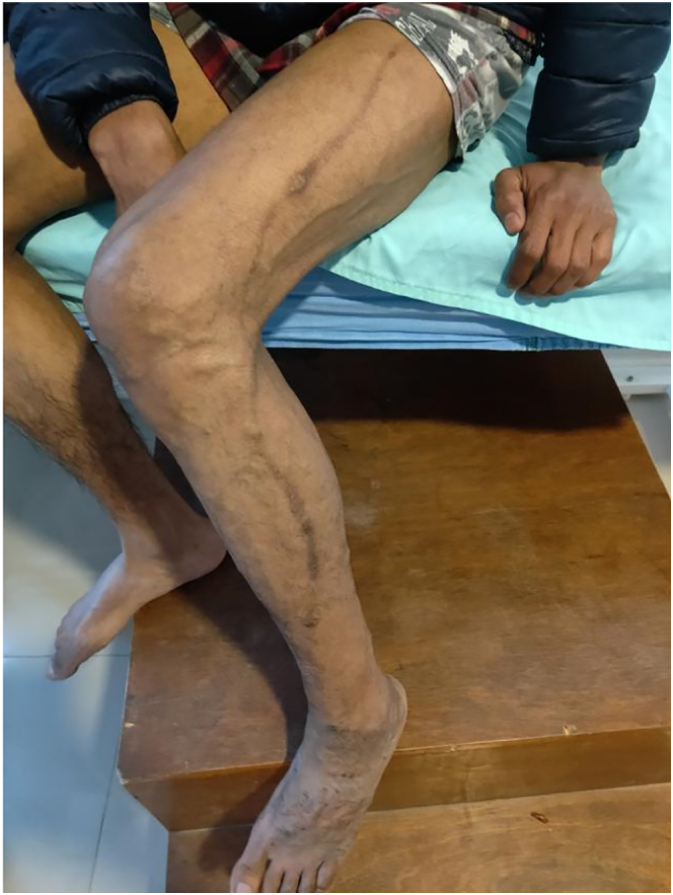
A two-year follow-up showing few residual varicosities which required sclerotherapy.

Electively Radio Frequency Ablation (RFA) of the lateral marginal vein of Servelle was done with adjunct sclerotherapy of the prominent veins and selected perforators. For RFA, cannulation was done in the distal calf and the RFA catheter could be negotiated well up to the proximal junction. A total of 11 segments of RFA was done with each treatment length of 7 cm, the temperature of 120 °C, and the time of the 20s. As shown in Fig. 2, Fig. 3, there was immediate improvement following RFA. For sclerotherapy 4 ml of inj. polidocanol mixed with 4 ml of normal saline and 4 ml of air via Tessari technique was used and given under ultrasonography guidance. Following the procedure, intraoperative Doppler ultrasonography didn't find significant residual varicose veins and thus segmental excision of the vein was not done. The patient was discharged the following day with analgesics and advised for compression bandaging for a month. In a follow-up of 40 days, the patient has much better cosmetic results and the pain has completely subsided. For residual varicosities, two more settings of sclerotherapy were done at three weeks and four weeks. In serial follow-up of two years with Doppler ultrasonography, there was the resolution of the lateral marginal vein along with the absence of incompetent perforators. Though there are some residual varicosities, for which sclerotherapy has been planned, other symptoms have completely subsided (Fig. 4).

## 3 Discussion

The lateral marginal vein is the embryonic persistence of a part of the superficial venous system which usually occurs in association with Klippel Trenaunay Syndrome [6], [7]. When the venous trunk fails to mature during the embryonic period, it remains as the lateral marginal vein after birth. It is a very rare condition. Normally, marginal vein almost always occurs along with lymphatic malformation as the most common occurrence in KT syndrome. These veins do not have valves like the varicose veins and they cause chronic venous insufficiency more than varicose veins. So, it carries a higher risk of DVT and PE and this is the most dangerous form of AV malformation. The weakness of smooth muscle of tunica media of the vessel wall and the lack of valve in the vein and reflux is the reason for the high risk of VTE and PE [8].

Mild symptoms include pain or asymptomatic prominent veins. This condition can also lead to leg length discrepancy, abnormal tortuous vein, erysipelas and ulcer, venous thrombosis, and pulmonary embolism. Varicose veins may cause venous ulceration after a prolonged period of time. Investigation of choice for KT syndrome is DUS besides clinical evaluation. Doppler is done in standing or supine position to assess reflux, patency of deep veins and presence of perforators. Further investigations like MRI or CT can be done to confirm the diagnosis and assess the extent.

Management basically is conservative. Surgical management is only done in rare situations and in selected cases. Choosing the cases for surgery is challenging due to excessive tortuosity and multiple perforators. Even with surgical intervention, the recurrence rate is very high, therefore, it is very wise to choose minimally invasive surgery with low recurrence and fewer complications. If the abnormal vein is causing significant problems due to pain, swelling, itchiness, pigmentation, ulceration, infection, enlargement of a limb involving one or both lower limbs etc., a surgical treatment modality is needed [4], [8].

Due to excessive tortuosity and multiple perforators open ligation and phlebectomy is usually preferred. Before surgery, venous anatomy must be confirmed with contrast phlebography, MRI, and MRP. Lymphaticovenular anastomosis has shown desirable results. Some cases have reported help from radiotherapy to induce regression of hemangioma. However, results can be slow to develop.

Endovenous laser therapy of the greater saphenous vein is gaining support for the management of varicosities in the general public and in patients with KTS. Debulking procedures have limited use and may damage venous and lymphatic structures, leading to increased oedema in the affected limb. Thus not preferred. It is important to find out that the patient has a normal functioning deep vein system before surgical intervention, otherwise there is a very high risk of deep vein thrombosis or venous hypertension due to the absence of a deep vein system [4].

Some literature contraindicated the use of the Endovenous ablation (EVA) technique due to the high risk of VTE [9], [10]. However, RFA has been reported to have great patient satisfaction as there is decreased pain and complications compared to classical surgical excision [11], [12], [13]. In our case, one of the main dilated veins was not very tortuous and it seemed RFA catheter could be negotiated. Moreover, the post RFA outcome was satisfactory.

Following the procedure, good results in our case are because RFA ablated the straight dilated venous segment. Postoperative sclerotherapy with 0.75% to 1% polidocanol or sodium tetradecyl sulfate (Sotradecol) has been reported to be a useful adjunct to surgery in patients with intractable symptoms [4].

## 4 Conclusion

Varicosities of the lateral marginal vein of Servelle can be managed successfully by radiofrequency ablation and adjunct sclerotherapy in selected cases.

## Consent

Written informed consent was obtained from the patient for publication of this case report and accompanying images. A copy of the written consent is available for review by the Editor-in-Chief of this journal on request.

## Provenance and peer review

Not commissioned, externally peer-reviewed.

## Ethical approval

N/A.

## Funding

None.

## Guarantor

Robin Man Karmacharya.

## Research registration number

N/A.

## CRediT authorship contribution statement

Conceptualization; Investigation; Methodology; Project administration; Resources: Robin Man Karmacharya, Kushal Gautam, Sushil Dahal
Supervision: Robin Man Karmacharya, Satish Vaidya
Roles/Writing - original draft; Writing - review & editing: Sushil Dahal, Robin Man Karmacharya, Satish Vaidya, Kushal Gautam, Swechha Bhatt, Niroj Bhandari.

## Declaration of competing interest

None.
